# ODs with a positive TPR conclusion, not subject to a conditional approval, and approved without requering a pass would be more likely to be reimbursed in Spain

**DOI:** 10.1186/s13023-022-02610-4

**Published:** 2023-01-06

**Authors:** José Luis Poveda, Claudia Gómez, Alicia Gil, Xavier Badia

**Affiliations:** 1 Hospital Universitario y Politécnico de La Fe, Valencia, Spain; 2 Omakase Consulting S.L., Barcelona, Spain

**Keywords:** Orphan drugs, Regulatory Variables, Pricing, Reimbursement, Spain

## Abstract

**Background:**

The present study aims to assess clinical and regulatory variables that would influence pricing and reimbursement (P&R) decisions for Orphan Drugs (ODs) in Spain. ODs approved by the European Commission (EC) between 2006 and 2021 were classified according to their P&R status in Spain: approved, undergoing decision and rejected. A statistical analysis was carried out to assess the potential association between clinical and regulatory variables and P&R decision of ODs in Spain: therapeutic area, rarity of disease, existence of alternative therapies, availability of survival-related outcomes, safety profile, type of population, conditional approval status granted by the European Medicines Agency (EMA) and a positive Therapeutic Positioning Report (TPR) opinion.

**Results:**

111 ODs have been approved by the EC and have obtained marketing authorisation in Spain between 2006 and 2021. Out of the 111 ODs, 57 (51.4%) were reimbursed, 24 (21.6%) were undergoing decision and 30 (27%) were rejected. According to the statistical analysis, ODs with a positive TPR conclusion (*p-*value < 0.01), not subject to a conditional approval by the EMA (*p-*value < 0.05) and approved without the obligation to conduct a post-authorisation safety study (PASS) (*p-*value < 0.05)*,* were statistically significant, and therefore, would be more likely to obtain P&R approval in Spain.

**Conclusions:**

This study shows that the TPR plays a key role in the P&R process in Spain and highlights that traditional evaluation tools, such us safety and efficacy, were the main drivers of P&R decisions for ODs. A positive conclusion of the TPR, non-conditional approval by the EMA and no obligation for a PASS seems to favourably affect P&R decisions in Spain.

## Background

More than 30 million inhabitants in the European Union (EU) suffer from a rare disease (RD) [1]. Although there is no universal definition for RDs [2], in the EU they are defined as those affecting no more than 5 per 10,000 inhabitants, with none or limited choice of therapeutic options. Some of these conditions are extremely rare or ultrarare, affecting less than 1 per 50,000 inhabitants [3]. Despite their low prevalence, they are life-threatening or chronically debilitating conditions with a high burden and very often limited level of awareness [1, 4]. RDs have a high impact on patients, their families, healthcare systems and even society in general, and are characterized by pain, disability, significant organ damage, and high mortality rates [5]. Although their prevalence is low, RDs are numerous and heterogeneous [6]. The true burden of rare diseases in Europe and elsewhere is difficult to estimate, since epidemiological data for most of these diseases are not available. It is estimated that more than 6000 RDs exist [1], affecting between 6 and 8% of the population.

RDs were so called “orphans” because they were neglected for many years. Orphan Drugs (ODs) are those intended to diagnose, prevent, or treat RDs [3]. RDs are now a public health priority within the European legislation. The EU Council established that patients suffering from rare conditions should be entitled to the same quality of treatment as patients suffering from more prevalent conditions. With that purpose, the EU introduced specific incentives for companies to develop OD to treat RDs, to compensate for the small market size and, introducing specific guidelines and requirements for clinical development programmes to reduce the uncertainty to develop an OD [4]. Applications for orphan designation are evaluated by the European Medicines Agency's (EMA) Committee for Orphan Medicinal Products (COMP). Once the product has been authorised by the European Commission (EC), ODs must be nationally authorised by local authorities at each member state before entering the market [7]. Pricing and reimbursement (P&R) decisions for ODs are determined at the national level, under varying evaluation and decision-making contexts which can often result in differences in restrictions and access levels for patients across different territories [8].

The distinctive features of ODs—limited knowledge and heterogeneity of the diseases, the limitations in following “standard” clinical trial development programmes due to small and typically heterogeneous patient populations, and the lack of hard clinical endpoints [9] pose an additional challenge in the appraisal of these products [10].

In Spain, the Committee on Pricing of Medicines, and Healthcare Products (CIPM), responsible for the final P&R decision, includes in their P&R resolutions the criteria used to justify such decisions. However, information on how these criteria are either measured or defined is not provided [11]. Therefore, the drivers influencing the approval or denial of a drug P&R are not clear [12], which could be interpreted as the existence of other factors influencing the P&R decisions within the Spanish NHS.

To reinforce decision-making, the Therapeutic Positioning Report (TPR) was introduced in Spain in 2013. Despite its name, the TPR is conditioned to the P&R negotiation and the final positioning of a new drug comes after the Directorate-General for the Basic Portfolio of Services of the National Healthcare and Pharmacy System (DGCBF) issues the reimbursement decision (and price). In a previous study, the impact of the TPR conclusion in the P&R process in Spain was reported, demonstrating its key role [13]. In 2020, the Consolidation Plan for the TPR was launched. To that end, a new Drug Evaluation Network (REvalMed NHS) was established, integrating alliances between the DGCBF, the Spanish Medicines Agency (AEMPS) and the representatives of the Spanish Regions, embodied into seven therapeutic nodes. With the introduction of the REvalMed NHS process, the TPR formally integrates the economic evaluation to assess the cost-effectiveness and/or budget impact of the new drug in the Spanish National Healthcare System (NHS) [14].

In Spain, once the companies submit the reimbursement request for a new medicine, they only have the possibility to discuss with the Ministry of Health during the allegation of the TPR, and during the P&R negotiation with DGCBF.

As a next step of the previous work [13], this study aims to review and assess the clinical and regulatory variables that might be relevant for the reimbursement decision of ODs in Spain.

## Methods

ODs approved by the EC and granted marketing authorisation in Spain were identified and stratified according to their reimbursement status. Then, relevant variables that could influence the P&R process in Spain were selected and study’s hypotheses were defined accordingly. Finally, a regression analysis was performed to test the validity of these hypotheses and to assess which variables influence the P&R process in Spain.

### Identification of orphan drugs approved by the European Commission with Spanish marketing authorisation, and their reimbursement status

Medicines with current orphan designation by the COMP and authorised by the EC until 2021 were identified. This information was extracted from the Community Register of Orphan Medicinal Products [15]. In a second step, information on marketing authorisation granted by the AEMPS and authorisation dates were retrieved from the Spanish Medicine Online Information Centre (CIMA) search engine [16]. The Spanish marketing authorisation dates granted by the AEMPS were used to analyse evaluation timelines (months) from Spanish marketing authorisation to P&R decision date. Eventually, the BIFIMED database was used to search for reimbursement status of each OD authorised in Spain until 2021 [17]. ODs were classified as “approved” (ODs that have had their P&R request approved), “under P&R decision process” (ODs for which P&R had been requested but are still under review/ negotiation), and “rejected” (ODs that have seen their P&R request rejected).

### Identification and description of clinical and regulatory variables relevant for the price and reimbursement process of orphan drugs in Spain

The variables considered for the analysis resulted from the official P&R criteria established by the Royal Decree Law 1/2015 of 24 July to evaluate the inclusion of new drugs [11], as well as from the variables reported in the mandatory information that the Marketing Authorisation Holder (MAH) must provide to European and Spanish regulatory bodies as a step into the centralised authorisation process and national P&R decision. Information was retrieved from ODs clinical trials, from their respective European Public Assessment Reports (EPARs) [18], or from the TPRs on the AEMPS website [19]. When information could not be found in these documents, a search in PubMed and grey literature was conducted. In addition, these identified clinical variables were tested in previous phases of this study: *(i) Therapeutic area, (ii) Outcomes classification*, *(iii) Therapeutic alternatives, (iv) Rarity of disease, and (vi) Type of population.* Regarding regulatory variables*, (i) TPR conclusion* and (*ii) Conditional approval* were included*.* Table 1 shows how these variables were defined and classified for the analysis. For those ODs without TPR, reimbursed ODs were considered to have a positive TPR conclusion. Conversely, ODs with a rejected P&R decision were considered to have a questionable opinion with respect to the EMA resolution as a TPR conclusion.

**Table 1:** Definition and classification of the variables relevant for the price and reimbursement process in Spain

Variable	Definition	Classification
*Clinical variables*		
(i) Therapeutic area	Therapeutic area was defined according to the ATC code [35]	Oncologic ODs Other
(ii) Outcomes classification	Each ODs clinical trials were analysed and classified depending on the outcomes used in the pivotal study: survival-related outcomes (i.e. overall survival) vs other outcomes (PROs, biomarkers, etc.) [36]	Survival Other
(iii) Therapeutic alternatives	ODs with therapeutic alternatives were defined as those intended to treat the same indication	Yes No
(iv) Rarity of disease	Rarity was defined according to the prevalence of the disease for which ODs were indicated. Rare diseases affect < 5/10000 inhabitants and ultra-rare diseases affect < 1/50000 [3]	Rare Ultra-rare
(v) Safety profile	Safety was defined according to the obligation by the EMA to carry out a PASS to obtain further information on a medicine’s safety [37]	PASS No PASS
(vi) Type of population	Type of population refers to whether ODs are intended to treat paediatric patients or not, defined as those patients under 18 years of age	Paediatric Other
*Regulatory variables*		
(i) TPR conclusion	The TPR conclusion was extracted from a positive opinion in the TPR (new drug recommended to a group of patients or equivalent to approved alternatives) or a questionable opinion with respect to the EMA [19]	Positive Questionable
(ii) Conditional approval	ODs with conditional approval granted by the EMA were defined as those with a positive benefit-risk balance but requiring clinical studies that have not yet been completed [38]	Yes No

*ATC* Anatomical, Therapeutic, Chemical classification system, *EMA* European Medicines Agency, *ODs* Orphan Drugs, *PASS* post-authorisation safety study, *PRO* Patient-Reported Outcomes, *TPR* Therapeutic Positioning Report

Study hypotheses were defined for the variables that could have an impact on P&R decisions. ODs would be more likely to be reimbursed if they were (i) indicated for oncologic diseases, (ii) based on survival-related outcomes, (iii) lacking other therapeutic alternatives, (iv) without an obligation to conduct a post-authorisation safety study (PASS), (v) intended to treat ultra-rare diseases, and (vi) indicated for paediatric patients. ODs with a (i) positive conclusion in the TPR and (ii) ODs without conditional approval granted by the EMA would also be more likely to be reimbursed.

### Statistical analysis

Approved ODs by the EC and granted Spanish marketing authorisation until 2021 were included in the analysis and categorised by their P&R status. Descriptive statistics were performed for quantitative variables (including time from Spanish marketing authorisation to P&R decision) and qualitative variables (clinical and regulatory variables). Mean (± SD) values were calculated for evaluation timelines. Frequency tables were displayed to describe data from clinical and regulatory variables.

As the study aimed to identify the variables that might positively influence the reimbursement decision of ODs in Spain, a Binary Dependent Variable (BDV) Model was considered for *reimbursement status* (Eq. 1). Therefore, only ODs with approved or rejected P&R were included in the regression analysis, excluding ODs under P&R decision process.

### Binary dependent variable (BDV) model

1 $$y = \left\{ {\begin{array}{*{20}l} 0 \hfill & {if\;rejected} \hfill \\ 1 \hfill & {if\;approved} \hfill \\ \end{array} } \right.$$

First, bivariate analyses were carried out using the χ2 test of association between the dependent variable (reimbursement status, stratified by approval or rejected) and the independent variables (clinical and regulatory variables) [20, 21]. Then, a logistic regression model was used to test the validity of the hypotheses defined for the identified ODs [22]. All statistical analyses were conducted on the statistical software Stata/IC15 [23].

## Results

### Orphan drugs authorised in Spain and approved by the European Commission from 2006 to 2021 and description of their reimbursement status in Spain

A total of 128 ODs have been approved by the EC between 2006 and 2021. Of those, 111 (86.7%) had been granted marketing authorisation in Spain, from which 57 (51.4%) had received P&R approval, 24 (21.6%) were undergoing the P&R process, and 30 (27%) had been rejected (Table 2). Mean time from Spanish marketing authorisation to P&R approval was 18.6 ± 11.9 months, with a minimum of 3 months (Kymriah® and Trepulmix®) [24, 25] and a maximum of 52 months (Revestive®) [26]. Mean time from marketing authorisation to P&R rejection was 17.6 ± 8.4 months. Before the inclusion of the TPR in 2013, the mean time from P&R request to P&R decision was 19.1 months; and after the inclusion of the TPR, the mean time was 18.2 months.

**Table 2:** List and description of identified variables for orphan drugs authorised in Spain from 2006 to 2021

Commercial name	P&R status	Therapeutic Area	Existence of therapeutic alternatives	Rarity of disease	Outcome	Safety	Type of population	TPR conclusion	Conditional approval
Nexavar®	Reimbursed	Oncologic	Yes	Rare	Survival	No	Other	Positive	No
Soliris®	Reimbursed	Oncologic	Yes	Rare	Other	No	Other	Positive	No
Vpriv®	Reimbursed	Other	Yes	Ultra-rare	Other	No	Other	Positive	No
Tobi Podhaler®	Rejected	Other	Yes	Rare	Other	No	Other	Negative	No
Votubia®	Reimbursed	Oncologic	No	Rare	Other	No	Other	Positive	No
Vyndaqel®	Reimbursed	Other	No	Ultra-rare	Other	Yes	Other	Positive	No
Xaluprine®	Under P&R decision process	Oncologic	Yes	Rare	Other	No	Other	Not published	No
Bronchitol®	Rejected	Other	Yes	Rare	Other	No	Other	Negative	No
Signifor®	Reimbursed	Other	Yes	Rare	Other	No	Other	Positive	No
Kalydeco®	Reimbursed	Other	No	Rare	Other	No	Other	Positive	No
Revestive®	Reimbursed	Other	No	Ultra-rare	Other	Yes	Other	Positive	No
Dacogen®	Reimbursed	Oncologic	Yes	Rare	Survival	No	Other	Positive	No
Adcetris®	Reimbursed	Oncologic	Yes	Ultra-rare	Survival	No	Other	Positive	Yes
NexoBrid®	Rejected	Other	No	Rare	Other	Yes	Other	Negative	No
Iclusig®	Reimbursed	Oncologic	No	Rare	Other	Yes	Other	Positive	No
Imnovid®	Reimbursed	Oncologic	Yes	Rare	Survival	Yes	Other	Positive	No
Procysbi®	Rejected	Other	Yes	Ultra-rare	Other	No	Other	Positive	No
Orphacol®	Reimbursed	Other	No	Ultra-rare	Survival	No	Other	Positive	No
Opsumit®	Reimbursed	Other	Yes	Ultra-rare	Survival	No	Other	Positive	No
Sirturo®	Rejected	Other	Yes	Rare	Other	No	Other	Positive	Yes
Cometriq®	Rejected	Oncologic	Yes	Ultra-rare	Survival	No	Other	Positive	Yes
Adempas®	Reimbursed	Other	No	Ultra-rare	Other	No	Other	Positive	No
Granupas®	Rejected	Other	No	Rare	Other	No	Other	Negative	No
Deltyba®	Reimbursed	Other	Yes	Rare	Other	No	Other	Positive	Yes
Vimizim®	Rejected	Other	No	Ultra-rare	Other	Yes	Other	Negative	No
Sylvant®	Reimbursed	Oncologic	No	Rare	Other	Yes	Other	Positive	No
Gazyvaro®	Reimbursed	Oncologic	Yes	Rare	Survival	No	Other	Positive	No
Translarna®	Rejected	Other	No	Rare	Other	No	Other	Negative	Yes
Scenesse®	Rejected	Other	No	Ultra-rare	Other	No	Other	Negative	No
Cerdelga®	Reimbursed	Other	Yes	Rare	Other	Yes	Other	Positive	No
Holoclar®	Rejected	Other	No	Rare	Other	No	Other	Positive	Yes
Strensiq®	Rejected	Other	No	Ultra-rare	Other	No	Paediatric	Positive	No
Farydak®	Rejected	Oncologic	Yes	Rare	Survival	No	Other	Negative	No
Kanuma®	Reimbursed	Other	No	Ultra-rare	Survival	Yes	Other	Positive	No
Raxone®	Rejected	Other	No	Rare	Other	No	Other	Negative	No
Cresemba®	Reimbursed	Other	Yes	Ultra-rare	Survival	No	Other	Positive	No
Kyprolis®	Reimbursed	Oncologic	Yes	Rare	Survival	No	Other	Positive	No
Blincyto®	Rejected	Oncologic	Yes	Rare	Survival	Yes	Other	Negative	No
Ravicti®	Reimbursed	Other	Yes	Rare	Other	Yes	Other	Positive	No
Wakix®	Reimbursed	Other	Yes	Rare	Other	Yes	Other	Negative	No
Idelvion®	Reimbursed	Other	Yes	Ultra-rare	Other	No	Other	Positive	No
Alprolix®	Reimbursed	Other	Yes	Ultra-rare	Other	No	Other	Positive	No
Darzalex®	Reimbursed	Oncologic	Yes	Rare	Survival	No	Other	Positive	No
Galafold®	Reimbursed	Other	Yes	Ultra-rare	Other	No	Other	Positive	No
Onivyde pegylated®	Reimbursed	Oncologic	Yes	Rare	Survival	No	Other	Positive	No
Ninlaro®	Rejected	Oncologic	Yes	Rare	Survival	No	Other	Negative	Yes
SomaKit TOC®	Reimbursed	Other	Yes	Rare	Other	No	Other	Positive	No
Ocaliva®	Reimbursed	Other	No	Ultra-rare	Other	No	Other	Positive	Yes
Cystadrops®	Rejected	Other	Yes	Ultra-rare	Other	No	Other	Positive	No
Ledaga®	Rejected	Oncologic	Yes	Rare	Other	No	Other	Positive	No
Chenodeoxycholic acid Leadiant®	Reimbursed	Other	No	Ultra-rare	Other	No	Other	Positive	No
Natpar®	Rejected	Other	No	Ultra-rare	Other	No	Other	Positive	Yes
Qarziba®	Under P&R decision process	Oncologic	Yes	Rare	Other	Yes	Other	Not published	No
Spinraza®	Reimbursed	Other	No	Ultra-rare	Survival	No	Other	Positive	No
Brineura®	Under P&R decision process	Other	No	Ultra-rare	Other	Yes	Other	Not published	No
Besponsa®	Reimbursed	Oncologic	No	Rare	Survival	No	Other	Positive	No
Oxervate®	Rejected	Other	No	Rare	Survival	No	Other	Negative	No
Xermelo®	Rejected	Other	Yes	Rare	Other	No	Other	Negative	No
Rydapt®	Reimbursed	Oncologic	Yes	Ultra-rare	Survival	No	Other	Positive	No
Lutathera®	Reimbursed	Other	Yes	Ultra-rare	Survival	No	Other	Positive	No
Zejula®	Reimbursed	Oncologic	Yes	Ultra-rare	Survival	No	Other	Positive	No
Jorveza®	Rejected	Other	Yes	Rare	Other	No	Other	Positive	No
Prevymis®	Reimbursed	Other	Yes	Rare	Other	No	Other	Negative	No
Crysvita®	Reimbursed	Other	No	Ultra-rare	Other	Yes	Paediatric	Positive	Yes
Alofisel®	Reimbursed	Oncologic	No	Rare	Other	No	Other	Positive	No
Lamzede®	Rejected	Other	No	Ultra-rare	Other	No	Other	negative	No
Mylotarg®	Reimbursed	Oncologic	No	Rare	Survival	No	Other	Positive	No
Amglidia®	Rejected	Other	Yes	Ultra-rare	Other	No	Paediatric	Negative	No
Tegsedi®	Reimbursed	Other	Yes	Rare	Other	No	Other	Positive	No
Verkazia®	Rejected	Other	Yes	Rare	Other	No	Paediatric	Negative	No
Myalepta®	Rejected	Other	Yes	Ultra-rare	Other	No	Other	Positive	No
Kymriah®	Reimbursed	Oncologic	Yes	Rare	Survival	Yes	Other	Positive	No
Yescarta®	Reimbursed	Oncologic	Yes	Rare	Survival	Yes	Other	Positive	No
Vyxeos®	Rejected	Oncologic	Yes	Rare	Survival	No	Other	Positive	No
Mepsevii®	Reimbursed	Other	No	Ultra-rare	Other	No	Other	Positive	No
Onpattro®	Reimbursed	Other	Yes	Rare	Other	No	Other	Positive	No
Cablivi®	Reimbursed	Other	Yes	Ultra-rare	Other	No	Other	Positive	No
Symkevi®	Reimbursed	Other	Yes	Rare	Other	No	Other	Positive	No
Takhzyro®	Reimbursed	Other	Yes	Ultra-rare	Other	No	Other	Positive	No
Luxturna®	Reimbursed	Other	No	Rare	Other	Yes	Other	Positive	No
Poteligeo®	Reimbursed	Oncologic	Yes	Rare	Survival	No	Other	Positive	No
Namuscla®	Rejected	Other	No	Rare	Other	No	Other	Negative	No
Palynziq®	Rejected	Other	No	Rare	Other	Yes	Other	Negative	No
Waylivra®	Under P&R decision process	Other	No	Ultra-rare	Other	Yes	Other	Not published	No
Trecondi®	Under P&R decision process	Oncologic	Yes	Rare	Other	No	Other	Not published	No
Epidyolex®	Reimbursed	Other	Yes	Ultra-rare	Other	No	Other	Positive	No
Xospata®	Rejected	Oncologic	No	Rare	Survival	No	Other	Negative	No
Isturisa®	Under P&R decision process	Other	Yes	Ultra-rare	Other	No	Other	Not published	No
Polivy®	Reimbursed	Oncologic	Yes	Rare	Other	Yes	Other	Positive	Yes
Givlaari®	Reimbursed	Other	Yes	Ultra-rare	Other	No	Other	Positive	No
Trepulmix®	Reimbursed	Other	Yes	Rare	Other	No	Other	Positive	No
Zolgensma®	Reimbursed	Other	Yes	Ultra-rare	Other	Yes	Paediatric	Positive	Yes
Reblozyl®	Under P&R decision process	Other	Yes	Rare	Other	No	Other	Not published	No
Daurismo®	Under P&R decision process	Oncologic	Yes	Rare	Survival	No	Other	Not published	No
Hepcludex®	Under P&R decision process	Other	No	Rare	Other	No	Other	Not published	No
Kaftrio®	Reimbursed	Other	Yes	Rare	Other	Yes	Other	Positive	No
Blenrep®	Under P&R decision process	Oncologic	Yes	Rare	Survival	No	Other	Not published	Yes
Idefirix®	Under P&R decision process	Oncologic	No	Rare	Other	Yes	Other	Not published	Yes
Adakveo®	Under P&R decision process	Other	No	Rare	Other	Yes	Other	Not published	Yes
Oxlumo®	Under P&R decision process	Other	No	Rare	Other	Yes	Other	Not published	No
Tecartus®	Under P&R decision process	Oncologic	Yes	Rare	Survival	No	Other	Not published	Yes
Libmeldy®	Under P&R decision process	Other	No	Rare	Survival	No	Paediatric	Not published	No
Fintepla®	Under P&R decision process	Other	Yes	Rare	Other	Yes	Other	Not published	No
Inrebic®	Under P&R decision process	Oncologic	Yes	Rare	Survival	Yes	Other	Not published	No
Pemazyre®	Under P&R decision process	Oncologic	No	Rare	Survival	No	Other	Not published	Yes
Evrysdi®	Under P&R decision process	Other	Yes	Rare	Survival	No	Other	Not published	No
Koselugo®	Under P&R decision process	Oncologic	No	Rare	Survival	Yes	Paediatric	Not published	Yes
Enspryng®	Under P&R decision process	Oncologic	Yes	Rare	Other	No	Other	Not published	No
Bylvay®	Under P&R decision process	Other	No	Ultra-rare	Other	No	Other	Not published	No
Minjuvi®	Under P&R decision process	Oncologic	Yes	Rare	Survival	No	Other	Not published	Yes
Aspaveli®	Under P&R decision process	Oncologic	No	Ultra-rare	Other	Yes	Other	Not published	No

#### Relevant variables for the pricing and reimbursement process in Spain

Out of the 111 ODs with marketing authorisation in Spain, 41 (36.9%) were indicated for oncologic diseases, 43 (38.7%) were indicated for a disease with no therapeutic alternatives and 40 (36.0%) were indicated for ultra-rare diseases (< 1/50000 inhabitants). Thirty-seven (33.3%) ODs had a survival-related endpoint included in their pivotal study, and 81 (73.0%) did not have to conduct a PASS. Finally, 49 (44.1%) out of 111 ODs were indicated for paediatric patients. Regarding regulatory variables, 66 (75.9%) ODs had a positive TPR opinion and 92 (83.0%) did not have a conditional approval by the EMA.

#### ODs for which P&R had been approved

Out of the 57 ODs with P&R approval in Spain, 21 (36.8%) were oncologic, 18 (31.6%) did not have a therapeutic alternative and 25 (43.9%) reimbursed were indicated for ultra-rare diseases. A survival-related endpoint was the outcome variable used in clinical trials of 21 (36.8%) reimbursed ODs and 41 (72.0%) did not have the obligation to conduct a PASS. Finally, 23 (40.3%) ODs were indicated for paediatric patients. For the regulatory variables, 55 (96.5%) ODs had a TPR with a positive opinion, and 51 (89.5%) ODs did not have a conditional authorisation granted by the EMA.

#### ODs with rejected P&R

Out of the 30 rejected ODs, 7 (23.3%) were indicated for oncologic diseases. Almost half (n = 14, 46.7%) of the rejected ODs had no therapeutic alternatives. Ten ODs (33.3%) were indicated for ultra-rare diseases and only 7 (23.3%) of the rejected ODs had survival-related endpoints as study outcomes. Twenty-six (86.7%) ODs did not conduct a PASS for their safety assessment and 14 (46.7%) ODs had been indicated for paediatric patients.

Regarding regulatory aspects, 11 (36.7%) out the 30 rejected ODs in Spain had a TPR with a positive opinion and 24 (80.0%) had not been subject to a conditional authorisation.

### Statistical analysis of potential relationship between clinical and regulatory variables and reimbursement status of ODs in Spain

The statistical analysis was carried out to assess the potential association between clinical and regulatory variables and P&R status of ODs in Spain. In the bivariate analysis, TPR conclusion showed a statistically significant association with the reimbursement decision. The logistic regression model was fitted to estimate the probability of reimbursement explained by the analysed clinical and regulatory variables. For reimbursement status, only ODs with approved or rejected P&R were considered (n = 87). The logistic regression results are shown in Table 3. The pseudo R-squared obtained in the model was 0.472, therefore the model explained 47% of the variability in the dependent variable. Values from 0.2 to 0.4 indicate an excellent model fit [27]. According to these findings, ODs with a positive TPR conclusion (*p-*value < 0.01), ODs not subject to a conditional approval by the EMA (*p-*value < 0.05)*,* and ODs approved without the obligation to conduct a PASS (*p-*value < 0.05)*,* were statistically significant, and therefore, would be more likely to obtain P&R approval in Spain.

**Table 3:** Impact of clinical and regulatory variables on the P&R approval according to logistic regression analysis

Variables		Coef	SE	t-value	*p*-value	[95% Conf Interval]		Sig
Therapeutic area	Oncologic	0.39	0.424	− 0.87	0.386	0.046	3.286	
Outcomes classification	Survival	3.134	3.381	1.06	0.29	0.378	25.961	
Therapeutic alternatives	No	0.823	0.58	− 0.28	0.782	0.206	3.279	
Rarity of disease	Ultra-rare	0.822	0.685	− 0.24	.814	0.16	4.209	
Safety	No PASS	0.055	0.068	− 2.34	0.02	0.005	0.627	**
Type of population	Paediatric	0.422	0.358	− 1.02	0.31	0.08	2.227	
TPR conclusion	Positive	225.762	304.687	4.02	0	16.028	3180.004	***
Conditional approval	No	9.07	8.729	2.29	0.022	1.375	59.818	**
	Constant	0.087	0.112	− 1.89	0.058	0.007	1.088	*
	Mean dependent var			0.655	SD dependent var			0.478
	Pseudo r-squared			0.472	Number of obs			87
	Chi-square			52.947	Prob > chi2			0.000
	Akaike crit. (AIC)			77.141	Bayesian crit. (BIC)			99.335

*SD* Standard deviation, *TPR* Therapeutic Positioning Report

***p < 0.01, **p < 0.05, *p < 0.1

## Discussion

The aim of the study was to assess clinical and regulatory variables that could influence the P&R decisions of ODs authorised in Spain.

From 2006 to 2021, 111 ODs had been granted marketing authorisation by the AEMPS, representing 86.7% of the total of ODs approved at the European level. However, only 51.4% (n = 57) of these ODs received P&R approval in Spain, 27% were rejected and 21.6% were undergoing the P&R decision process. This highlights that with the same evidence triggering EMA approval with or without conditions, the timing and level of access to ODs could vary across countries determined by differences in national criteria used for medicines assessments and P&R decisions [28].

Regarding evaluation timelines, the mean time from Spanish marketing authorisation to P&R decision after the inclusion of the TPR in 2013 was 18.2 months. P&R evaluation timelines have been slightly reduced since the inclusion of TPRs by an average of less than 1 month. This could be due to the fact that the performance of the TPR requires time. In addition, evaluation timelines could have been affected by the COVID pandemic over the last two years. Other reports that assessed the access to orphan medicines in Spain until December 2021 have reported similar findings in terms of estimated regulatory timelines during the P&R process, thus reinforcing the validity of the data presented in this study [29, 30].

Regression analysis showed that a positive TPR conclusion was key in the P&R decision in Spain. This is consistent with a previous study, where the association between a positive TPR and reimbursement of new ODs has been shown [13]. In addition, regression analysis has also shown that ODs whose evaluation is subject to less uncertainty, i.e. ODs without a conditional authorization by the EMA and without a PASS study, would be more likely to be reimbursed. Therefore, with respect to the findings discussed above, variables related to safety and efficacy have shown an impact on the likelihood of reimbursement. The study showed that traditional evaluation criteria were the main drivers in the P&R decision. A recent report by the Spanish Ministry of Health highlighted that clinical uncertainty (translated into financial uncertainty) actually increases the complexity in P&R decision-making [31]. Although the price of the ODs was not included in the analysis, we considered would be a key variable to explain reimbursement status, however, further studies would be needed to corroborate it. The prices for ODs may be higher, as it is difficult to recover the costs of innovation. Thus, ODs are unlikely to reach the standard cost-effectiveness thresholds [5, 32, 33]. However, many ODs are often reimbursed despite having incremental cost-effective ratios (ICERs) that are much higher than the willingness to pay (WTP). This suggests that, in practice, alternative approaches might be considered in ODs for P&R decisions, as the incorporation of new financing schemes reflected in the resolutions (e.g. expenditure cap, pharmacological protocols) [34].

Previous reports at national and international level have described similar findings on the identified ODs, their P&R status, and regulatory times to P&R decisions (e.g. aeLmhu report "Access to ODs in Spain”, Spanish Ministry of Health report "The evolution of the financing and pricing of ODs in the NHS”, and the “Waiting to Access Innovative Therapies (WAIT)” report performed by IQVIA) [29–31]. However, the main differentiating aspect of the present study is the assessment of the impact of TPR on P&R evaluation timelines and the assessment of clinical and regulatory variables that could be relevant in the P&R process of ODs in Spain.

Another finding to highlight from the regression analysis is that the absence of therapeutic alternatives does not seem to be associated with the P&R approval of an OD in Spain despite being a P&R criterion as established in article 92 of Royal Decree Law 1/2015 of 24 July. This could be due to the limitation of sample size, as despite having collected all available and published data, we still have a small sample size to be able to identify significant differences for some criteria in a multivariate analysis.

In addition, there are some variables, such as the authorisation under exceptional circumstances by EMA or the inclusion in the Valtermed registry in Spain, which have not been included in the study because the sample size is too small. In Spain, there are only 9 ODs approved under exceptional circumstances and with P&R resolution, and 11 ODs included to the Valtermed registry.

The study results have reflected the importance of the TPR prepared by the REvalMed NHS network in the reimbursement decision. However, unlike its name suggests, the final positioning of the drug is only established once the price has been negotiated with the DGCBF. Accordingly, the positioning of the drug is, among other factors, determined by its price. In addition, as stated in the above-mentioned report by Spanish Ministry of Health, clinical benefit uncertainty and price proposed by the MAH were highlighted as the main drivers to deny the P&R by the CIPM [31]. This would have been a determinant variable to have contributed to our analysis, but such information is not publicly available because official listed prices in the available databases do not reflect the reimbursement price agreed between the Ministry of Health and the MAH. Confidential prices would be around 40% of the list price, but they do not always follow the same pattern [13]. In addition, the reimbursed price depends on other variables such as the requested price, the price of other similar treatment alternatives and the medicine price in other EU reference countries, which are also not public and, therefore, not controllable.

Among the methodological limitations of the study, several assumptions were made. For those ODs appraised before the introduction of the TPR, it was assumed that reimbursed ODs had a positive TPR opinion. However, rejected ODs were assigned a questionable opinion of the TPR as opposed to the EMA's efficacy and safety assessment. Thus, we could include the maximum number of observations, with respect to the sample, in the analysis.

As the data cut-off point was December 2021, the number of observations to compare evaluation timelines before and after the introduction of REvalMed NHS in 2020 was not large enough (n = 9). Future analysis could assess the impact of the new procedure on evaluation timelines and reimbursement decisions.

As mentioned, economic criteria influencing the P&R decisions, such as the price of the OD and budget impact, have not been included in the statistical analysis, as the available official public prices do not reflect the reimbursement price. In addition, the study could have omitted alternative criteria considered by evaluators.

Other limitations come from the potential interaction between some of the explanatory variables. For instance, it could be assumed that ultra-rare diseases will present a limited arsenal of therapeutic alternatives. However, the objective of the model was to provide a construct of variables that could shed some light on P&R decisions in Spain. Considering the criteria established in Spain for the reimbursement of new drugs [11], it would be advisable to increase transparency regarding how these criteria are measured and assessed for decision-making [12] related to the value of a new drug.

## Conclusions

Out of the 111 ODs authorised by the AEMPS, 51.4% of these ODs received P&R approval in Spain until 2021, 27% were rejected and 21.6% were undergoing P&R decision. P&R approval would be associated with a positive TPR conclusion, non-conditional approval by the EMA and no obligation for a PASS. Therefore, the study highlighted the role that a TPR plays in the reimbursement process and showed that traditional evaluation tools, such us safety and efficacy, were the main drivers of P&R decisions for ODs. Although economic variables have not been included in the analysis, these are considered a decisive factor in the reimbursement process.

## Data Availability

In 2017, Omakase Consulting S.L. developed an OD database to collect data related to medicinal products with OD designation, currently authorised in Europe and their P&R situation in Spain. The datasets used and analysed during the current study are available from the corresponding author on reasonable request.

## Abbreviations

AEMPS: Spanish Medicines Agency
ATC: Anatomical, therapeutic, chemical classification system
BDV: Binary dependent variable
CIMA: Medicine Online Information Centre
CIPM: Committee on Pricing of Medicines and Healthcare Products
COMP: Committee for Medicinal Products and Orphan Products
DGCBF: Directorate-General for the Basic Portfolio of Services of the National Healthcare and Pharmacy System
EC: European Commission
EMA: European Medicines Agency
EPAR: European Public Assessment Report
EU: European Union
ICER: Incremental cost-effective ratio
MAH: Marketing authorisation holder
NHS: National Healthcare System
ODs: Orphan drugs
P&R: Price and reimbursement
PASS: Post-authorisation safety study
PRO: Patient-reported outcomes
RD: Rare disease
REvalMed: Drug evaluation network
SD: Standard deviation
TPR: Therapeutic Positioning Report
WTP: Willingness to pay
